# Dose-response relationship between lung function and chest imaging response to silica exposures in artificial stone manufacturing workers

**DOI:** 10.1186/s12940-024-01067-1

**Published:** 2024-03-02

**Authors:** Chi-Hsien Chen, Perng-Jy Tsai, Wen-Wen Chang, Cheng-Yao Chen, Chih-Yong Chen, Deborah Yates, Yue Leon Guo

**Affiliations:** 1 Department of Environmental and Occupational Medicine, College of Medicine and NTU Hospital, National Taiwan University (NTU), No. 7, Zhongshan S. Rd., Zhongzheng Dist, Taipei City, Taiwan; 2 Department of Environmental and Occupational Health, College of Medicine, National Cheng Kung University, 138 Sheng-Li Rd., North District, Tainan, 70403 Taiwan; 3 Division of Occupational Hazards Assessment, Institute of Labor, Occupational Safety and Health, Ministry of Labor, No. 99, Ln. 407, Hengke Rd., Xizhi Dist, New Taipei City, 221004 Taiwan; 4 Respiratory Medicine, St Vincent’s Public Hospital, Sydney, Australia; 5 St Vincent’s Hospital Clinical School, Sydney, Australia; 6 Institute of Occupational Medicine and Industrial Hygiene, National Taiwan University, No. 17, Xuzhou Rd., Zhongzheng Dist, Taipei City, Taiwan; 7 National Institute of Environmental Health Sciences, National Health Research Institutes, No. 35, Keyan Rd., Zhunan Township, Miaoli County, Taiwan; 8 Department of Environmental and Occupational Medicine, College of Medicine and National Taiwan University Hospital, National Taiwan University, Rm 339, 17 Syujhou Road, Taipei, 100 Taiwan

**Keywords:** Artificial stone, Exposure, Respiratory symptoms, Lung function, Chest CT, Diffusion capacity

## Abstract

**Background:**

Occupational exposure to artificial stone, a popular material used for countertops, can cause accelerated silicosis, but the precise relationship between silica dose and disease development is unclear.

**Objectives:**

This study evaluated the impact of silica exposure on lung function and chest imaging in artificial stone manufacturing workers.

**Methods:**

Questionnaire and spirometry assessments were administered to workers in two plants. A high-exposure subset underwent further evaluation, including chest CT and DLco. Weighting factors, assigned as proxies for silica exposure, were based on work tasks. Individual cumulative exposures were estimated using area concentration measurements and time spent in specific areas. Exposure-response associations were analyzed using linear and logistic regression models.

**Results:**

Among 65 participants, the mean cumulative silica exposure was 3.61 mg/m^3^-year (range 0.0001 to 44.4). Each 1 mg/m^3^-year increase was associated with a 0.46% reduction in FVC, a 0.45% reduction in FEV1, and increased lung function abnormality risk (aOR = 1.27, 95% CI = 1.03–1.56). Weighting factors correlated with cumulative exposures (Spearman correlation = 0.59, *p* < 0.0001), and weighted tenure was associated with lung function abnormalities (aOR = 1.04, 95% CI = 1.01–1.09). Of 37 high-exposure workers, 19 underwent chest CT, with 12 (63%) showing abnormal opacities. Combining respiratory symptoms, lung function, and chest X-ray achieved 91.7% sensitivity and 75% specificity for predicting chest CT abnormalities.

**Conclusion:**

Lung function and chest CT abnormalities occur commonly in artificial stone workers. For high-exposure individuals, abnormalities on health screening could prompt further chest CT examination to facilitate early silicosis detection.

**Supplementary Information:**

The online version contains supplementary material available at 10.1186/s12940-024-01067-1.

## Introduction

Artificial stone (AS), also known as engineered stone or quartz conglomerate, is a relatively new compound frequently used for kitchen and bathroom countertops due to its aesthetic appeal and lower cost compared with natural stone [1, 2]. AS is produced by solidifying a mixture of crushed stone and resin through a heating process. It typically contains more than 90% silica, significantly higher than natural stones such as marble (3%) and granite (30%) [3]. The processes involved in manufacturing, fabricating, and in-home installation of AS slabs, including mixing, cutting, grinding, polishing, and drilling, all result in workers being exposed to high levels of respirable crystalline silica (RCS).

Since 2010, AS-related silicosis cases have emerged globally, including in Spain [4–10], Israel [11, 12], Italy [10, 13], Australia [10, 14–19], Belgium [20], the United States [10, 21–24], and China [25, 26]. Respiratory surveillance programs have uncovered a high prevalence of silicosis among AS workers, with rates as high as 55% [5] and 21% [9] in Spain, 12% [16] and 28.2% [27] in Australia, and 12% in the United States [24]. In Israel, AS-related silicosis has increasingly required lung transplantation [11]. Numerous cases have demonstrated disease onset occurring within 10 years of exposure, indicating accelerated silicosis [1, 6–10, 17, 22, 25, 26]. Follow-up studies have documented a rapid decline in lung function [1]. Progressive deterioration of lung function and chest imaging have been observed even after cessation of exposure [8]. It remains unclear whether the high concentration of silica itself or interactions with metallic (pigments) [28] or organic (e.g., resin and curing agents) [29] components lead to a more aggressive disease course compared to natural stone silicosis. Based on prior pathological studies in coal miners, exposure to high concentrations of silica continue to be a pivotal factor in the development of rapidly progressive pneumoconiosis [30].

In the past five years, three cases of AS-related accelerated silicosis have been reported to the Occupational Injury and Disease Reporting System in Taiwan (https://nodis.osha.gov.tw/). Of these cases, one patient underwent a successful lung transplant, while another died from early infection following transplantation. The third patient passed away while waiting for a suitable donor. These sentinel cases highlighted the pressing need for a respiratory surveillance or case-finding program in Taiwan, as well other countries using artificial stone.

The evaluation of respiratory symptoms, spirometry, and chest X-ray (CXR) are standard approaches used in the health surveillance of individuals exposed to dust. Given the superior sensitivity of low-dose chest computed tomography (CT) compared to CXR in silicosis detection, this has been recommended as a valuable addition for the early silicosis detection in high-risk populations [31, 32]. Health screenings for AS workers in Australia has been updated to incorporate chest CT scans, because studies have confirmed that 43% of diagnosed silicosis cases will have normal CXR findings [17]. Chest CT scans can detect early-stage silicosis, such as category 0 in the International Labour Organization (ILO) classification, even when CXR is normal. A Spanish study tracking silicosis patients four years after exposure cessation demonstrated a gradient of progression to progressive massive fibrosis (PMF) between different ILO categories. Categories 0 to 3 developed PMF in 6.3%, 22.2%, 47.4%, and 66.7% of cases respectively over a period of four years, highlighting the need for early diagnosis [8]. However, defining at-risk populations and determining when to implement chest CT scanning have not been standardized. The Queensland official guideline considers workers who have been performing dry cutting for more than one year or wet cutting for more than three years as at high risk, indicating the need for chest CT [33]. Employment duration may not fully capture risk differences resulting from different cumulative exposures. To date, no study has yet linked actual RCS concentrations with lung function or chest imaging changes in AS workers. Addressing this knowledge gap should facilitate advances in health prevention and add to the evidence base of respiratory surveillance programs [3]. Therefore, we conducted a field study at two AS manufacturing plants in Taiwan to investigate the relationship between RCS exposure concentrations and the development of respiratory disease.

## Methods

### Design and study population

In October 2022, we conducted a cross-sectional study on the respiratory health of workers at two artificial stone manufacturing plants (Plant A and B). To our knowledge, there were at least five artificial quartz stone manufacturing plants in Taiwan. Following a recent case of reported silicosis at one of these manufacturing plants, we invited this particular plant and a neighbouring plant to participate in our study. These industries employ 68 individuals. During the invitation process, all employees were invited to participate and were given up to three weeks to consider their participation, during which we held an informational session to address any questions or concerns they might have about the study. Recruitment was done with company cooperation, and informed consent was obtained. Approved by the National Taiwan University Hospital’s Institutional Review Board, participants filled out a demographics, work history, and respiratory symptoms questionnaire and underwent pre-shift spirometry based on ATS/ERS standards. Workers were allowed to participate voluntarily, with the assurance that their decision would not affect their employment rights.

Owing to budget limitations, only 20 workers were provided with chest CT scans, CXR and tests for diffusion capacity for carbon monoxide (DLco) at a tertiary hospital. Initially, 20 workers from high-exposure levels were invited, including Raw Material Operator, Cutting Machine Operator, Grinding Machine Operator, and Vacuum Press Machine Operator. However, when one invited Grinding Machine Operator declined the hospital-based assessments due to concerns about radiation exposure, we extended an invitation to an administrative supervisor from the Grinding Machine Operation department instead.

For this study, a time compensation of 100 New Taiwan Dollars (approximately 3.3 US Dollars) was offered to those participating in the on-site questionnaire and lung function tests. Additionally, workers participating in the hospital-based assessments were provided with an extra 100 New Taiwan Dollars as a transportation allowance.

### Questionnaire assessment of job exposure and health

We used a structured questionnaire to evaluate smoking habits, respiratory symptoms [34], and job details. The questionnaire, based on Glass et al.‘s study [19], assessed time spent handling artificial stone and performing dry work, assigning weighting factors (WF) to responses as proxies for RCS dust exposure. Options ranged from “All artificial stone” (WF = 1) to “All natural stone” (WF = 0.3) and from “Never” (WF = 1) to “Always” (WF = 10) for dry operations, reflecting their silica content and dust generation. Weighted tenure, combining WFs and job duration, estimated cumulative exposure.

### Assessment of exposure to respirable crystalline silica

Two occupational physicians and two industrial hygienists conducted a factory walkthrough to identify distinct manufacturing processes and work areas for RCS exposure assessment. These areas included:

1. Raw Material Unloading: Unloading raw materials like quartz, resin, and additives.
2. Resin Premixing: Mixing resin with curing and coupling agents.
3. Artificial Quartz Sand Mixing: Blending resin mixture with quartz sand and powder.
4. Material Spreading and Pattern Application: Spreading mix onto molds and pattern drawing.
5. Vacuum Press: Subjecting mix in moulds to vacuum compression to form solid slabs.
6. Curing: Curing compressed slabs to solidify and ensure mechanical properties.
7. Cooling: Cooling cured slabs post-mould removal.
8. Cutting: Cutting cooled slabs as per customer specifications.
9. Back Grinding: Grinding cut slabs to desired thickness.
10. Front Grinding: Further grinding slabs, varying by machinery and speed.
11. Polishing: Polishing ground slabs for a smooth finish.
12. Quality Control: Inspecting polished slabs for appearance and quality.

Plant A, being larger than Plant B, encompasses all the aforementioned manufacturing processes. In contrast, Plant B is limited to the cutting and grinding processes. We combined workers from similar exposures into similar exposure groups. For each area, 3–6 sampling points were established to collect respirable dust samples, analysed using X-ray diffraction for the weight concentration of crystalline silica. Personal exposure concentrations were calculated based on the time spent in these areas and expressed as an 8-hour time-weighted average (TWA). Cumulative exposure was determined by multiplying individual exposure levels (8-hour TWA) by tenure, resulting in units of mg/m^3^-year.

### Assessment of forced lung function

Following American Thoracic Society guidelines [35], participants completed spirometry tests in a seated position, performing at least three forced expiratory manoeuvres for consistent and smooth flow-volume loops with less than 5% or 150 ml disparity in lung volume between the optimal two attempts. Key measures included forced vital capacity (FVC), forced expiratory volume in one second (FEV_1_), and the FEV1/FVC ratio, with the spirometer (SpiroTube, Thor Medical Systems, Hungary) calibrated using a 3 L syringe. Spirometric results were classified as obstructive with an FEV1/FVC ratio below the lower limit of normal (LLN), and restrictive if FVC was below LLN with a normal FEV1/FVC ratio [36]. Predicted FVC, FEV1, and LLN for FEV1/FVC and FVC were based on the Global Lung Initiative 2012 equation, incorporating ethnic adjustments for South East Asians [37]. Although employing a fixed FEV1/FVC cut-off value of 0.7 may lead to the underdiagnoses of chronic obstructive pulmonary disease in individuals younger than 50 years [38], in our data analysis, we utilized additional clinically common threshold values (0.75 and 0.7) to evaluate the impact of exposure on the occurrence of obstructive ventilatory impairment. DLco measurements were conducted at a hospital according to international recommendations [39] using the single-breath method with methane as the tracer gas, with participants abstaining from smoking on the test day. Impairment in diffusion capacity was characterized by a DLco value below the lower limit of normal (LLN) [36], which was calculated using the equation provided by the Global Lung Initiative 2017 equation [40].

### Chest imaging

During their hospital visit, participants received a standard chest radiograph and a low-dose helical chest CT scan. Initially, the images were independently interpreted by a physician specializing in occupational medicine and a pulmonologist. Subsequently, both specialists collaborated to code the findings using the International Classification of High-Resolution Computed Tomography for Occupational and Environmental Respiratory Diseases (ICOERD) criteria. Sum grades of six lung zones (upper, middle, and lower of each lung) were calculated for well-defined rounded opacities (RO), linear and/or irregular opacities (IR), ground-glass opacities (GGO), and emphysematous changes (EM).

### Statistical analysis

Participants were divided into high and low-exposure groups using median dust exposure values. Group differences in demographics and respiratory symptoms were analyzed with Chi-square, Fisher’s exact, or Mann-Whitney tests. Correlation between weighted tenure and dust exposure concentration was assessed via Spearman’s analysis, and exposure group consistency was checked using chi-square. Logistic regression evaluated the link between exposure and lung function impairment, while linear regression examined the exposure-response relationship with pulmonary parameters like FVC and FEV1 percentages. Adjustments were made for factors, including age, sex, body mass index, educational attainment, current smoking habits, past smoking habits, and cumulative smoking amount in pack-years. Educational attainment was included in the model adjustment to account for potential socioeconomic factors [41] that might influence lung function [42] and chronic respiratory diseases [43].

Sensitivity analysis was conducted by running several models with varying combinations of covariates and by excluding individuals with any history of smoking. This approach was taken to assess the robustness and reliability of the study’s findings, ensuring that conclusions are consistent across different model specifications.

Sensitivity and specificity were computed to assess the diagnostic accuracy of several assessment tools, specifically respiratory symptoms, spirometry, CXR, and DLco, in detecting opacities defined as an ICOERD sum score of RO, IR, GGO, and EM of 1 or greater [44], as identified by chest CT. A positive indication for respiratory symptoms was assigned if the response to any question regarding respiratory symptoms was “yes.” Additionally, the diagnostic accuracy of different combinations of assessment tools was analysed. In these combinations, a positive result was determined if any of the methods identified an abnormality.

The statistical significance threshold was set at a *P* value of less than 0.05.

## Results

Of the 68 workers invited to participate in the study, 65 provided their consent (57 from Plant A, 8 from Plant B). Among the three individuals who declined participation, all were employed at Plant A; one held an administrative staff position, while the remaining two were engaged in sales roles. The administrative staff member declined due to recent health examination and lacking of respiratory symptoms. The two sales persons refused to participate and attributed their non-participation to schedule conflicts between the health assessments and their outing for business. Table 1S summarizes the demographics and health conditions of workers from both plants, showing no significant differences in demographics or tenure, except for a lower proportion of higher-educated workers at Plant B, due to more administrative staff at Plant A. Health outcomes, including respiratory symptoms, lung function abnormalities, and chest CT results, were also similar across both plants.

The mean age of the participants was 42 years. They were divided into nine groups based on job titles and work patterns. Table 1 shows respirable silica dust exposure and weighting factors for each group. Raw Material Operators, handling material unloading and mixing, had the highest exposure. Vacuum Press Machine Operators primarily managed the press and assisted in other areas like material spreading, pattern application area, curing area, and cooling. Cutting Machine Operators worked with waterjet cutters and helped in cooling, while Grinding Machine Operators used water-suppressed grinding or polishing machines. Facility Management maintained factory machinery, Operations Supervisors oversaw operations, Quality Control conducted product inspections in the end-of-line area, and Administration and Research and Development staff worked in offices.

**Table 1:** The distribution of respirable free silica dust exposure and assignment of weighting factors among workers with various job titles

Job title		RCS level (mg/m^3^)*			Weighting factor		
	Number	Mean	Minimum	Maximum	Mean	Minimum	Maximum
Administration	15	0.001	0.001	0.001	0.96	0.3	1.5
Research and Development	4	0.001	0.001	0.001	2.63	1	4.5
Quality Control	2	0.029	0.001	0.056	0.9	0.3	1.5
Vacuum Press Machine Operator	4	0.175	0.099	0.2	4.5	1.5	7.5
Facility Management	15	0.325	0.001	0.622	2.13	0.6	10
Grinding Machine Operator	8	0.449	0.168	0.767	4.88	3	7.5
Operations Supervisor	4	0.57	0.415	0.622	5	1	10
Cutting Machine Operator	3	0.587	0.587	0.587	3.92	0.75	8
Raw Material Operator	10	2.878	0.27	4.44	7.8	4	10

*RCS levels were expressed as an 8-hour time-weighted average (TWA).

**Fig. 1:**
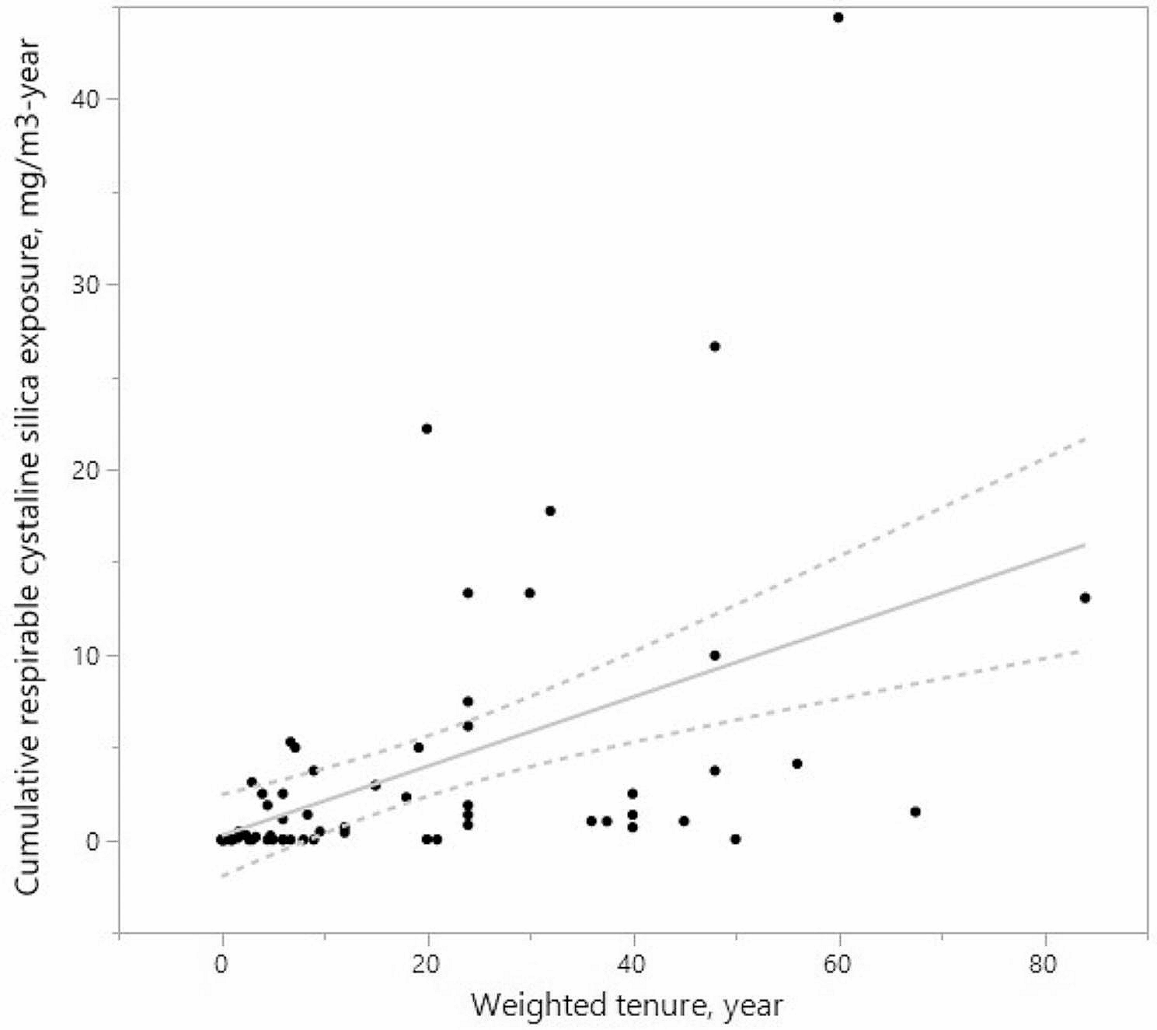
Scatter plot of weighted tenure in years (x-axis) and cumulative silica exposure in mg/m^3^-year (y-axis) among the study participants (Spearman’s correlation coefficient = 0.69, *P* < 0.0001). Each data point represents the cumulative exposure level of an individual worker. The regression line shows the positive association between weighted tenure and cumulative silica exposure (Estimated coefficient = 0.19, *P* < 0.0001)

Participants were divided into low and high exposure groups according to a median RCS exposure level of 0.168 mg/m³ (8-hour TWA). The low and high exposure groups were exposed to mean RCS concentrations of 0.02 mg/m³ and 1.12 mg/m³, respectively. The average cumulative exposures for these groups were 0.15 mg/m³-year and 6.23 mg/m³-year, respectively. For all participants, the mean cumulative exposure to RCS was 3.61 mg/m³-year, with a geometric mean of 0.22 mg/m³-year. The high exposure group had more male workers and lower education levels (Table 2). A strong correlation (Spearman correlation = 0.59, *P* < 0.0001) was found between questionnaire-derived weighting factors and RCS exposure, with a similar correlation noted for cumulative exposure, as illustrated in Figure 1. Classification concordance between RCS and weighting factors was high (Chi-square *P* < 0.0001), with 29 and 24 participants consistently categorized into high and low exposure groups in both methods.

**Table 2:** Comparison of basic characteristics, job exposure, and protective equipment usage between low and high respirable crystalline silica exposure groups

	Low exposure	High exposure	*P* value
	*n* = 28	*n* = 37	
Age, mean ± SD, yr	44.0 ± 14.0	39.9 ± 9.2	0.153
Male, n (%)	16 (57.1)	35 (94.6)	0.0003
Body mass index, mean ± SD	24.6 ± 4.2	26.6 ± 4.9	0.126
Education attainment > = 13 yrs, n (%)	23 (82.1)	18 (48.7)	0.006
Tobacco smoking, n (%)			0.051
Never	19 (67.9)	20 (54.1)	
Ex-smoker	0 (0)	7 (18.9)	
Current smoker	9 (32.1)	10 (27.0)	
Cumulative smoking amount, mean ± SD, pack*yr	9.1 ± 10.3	7.4 ± 8.5	0.699
Tenure, year, mean ± SD	6.0 ± 6.8	6.0 ± 4.3	0.989
Weighting factor, mean ± SD	1.3 ± 0.9	5.1 ± 3.2	< 0.0001
Weighting factor > median (2), n (%)	4 (12.1)	29 (87.9)	< 0.0001
Weighted tenure, mean ± SD, year	6.9 ± 10.1	26.6 ± 20.9	< 0.0001
Cumulative RCS exposure, mean ± SD, mg/m^3^-year	0.15 ± 0.33	6.23 ± 9.06	< 0.0001
Best respiratory PPE used at work			< 0.0001
No or regular flat mask, n (%)	3 (10.7)	1 (2.7)	
N95, n (%)	18 (64.3)	5 (13.5)	
Half-face mask, n (%)	2 (7.1)	10 (27.0)	
Full-face mask, n (%)	2 (7.1)	15 (40.5)	
PAPR, n (%)	3 (10.7)	6 (16.2)	
Fit test experience, n (%)	0 (0)	8 (21.6)	0.008

Abbreviations: PAPR, powered air-purifying respirator; PPE, personal protective equipment; RCS, respirable crystalline silica; SD, standard deviation

Cumulative smoking amount was only calculated for people ever having smoking habits

*p* values were calculated by for Chi-square Fisher’s exact test, or Mann-Whitney test

Regarding the usage of respiratory protective equipment, the high exposure group workers reported employing equipment with superior filtration efficiency. However, on-site visits revealed that only a small fraction of workers were correctly utilizing respiratory protective equipment (RPE). Only 22% of workers in the high exposure group reported having undergone fit testing for their RPE.

High exposure workers reported more chronic phlegm production (16% vs. 0% in low exposure, *P* = 0.033) and had lower FVC and FEV1, with more ventilatory impairments (32% vs. 4% in low exposure, *P* = 0.008) (Table 3). Multivariable logistic regression showed an increasing exposure-response relationship, with higher adjusted odds ratios (aOR) for greater exposure levels (aOR, 1.27; 95% CI, 1.03–1.56 per mg/m^3^-year for silica, and aOR, 1.04; 95% CI, 1.01–1.09 per year for weighted tenure) (Table 4). Using stricter FEV1/FVC ratio cut-offs (0.75 and 0.7) weakened the association between cumulative RCS exposure and obstructive ventilatory defects, with aORs of 1.05 (95% CI, 0.96–1.16) and 0.99 (95% CI, 0.77–1.28) per mg/m³-year, respectively, compared to the LLN approach (aOR, 1.07; 95% CI, 0.98–1.18 per mg/m³-year) (Table 4). Linear regression indicated that each 1 mg/m³-year increase in exposure reduced FVC and FEV1 by 0.44% (95% CI, -0.88–0.05) and 0.45% (95% CI, -0.81–0.08), respectively (Table 5). This effect size equates to a decrease of 24 ml in FVC and 20 ml in FEV1. Sensitivity analyses revealed that the results remained consistent, irrespective of whether the regression model was adjusted for educational attainment and smoking status (Tables 6 and 7). In the subgroup analysis of never smokers, the magnitude of the association between cumulative RCS exposure and obstructive ventilatory abnormalities diminished (aOR, 1.00; 95% CI, 0.88–1.22 per mg/m^3^-year).

**Table 3:** Pre- and post-shift lung function in laminators and non-laminators

	Low exposure	High exposure	*P* value
	*n* = 28	*n* = 37	
Respiratory symptoms, n (%)			
Morning cough in winter	6 (21.4)	7 (10.8)	0.306
Coughing throughout the day in winter	8 (28.6)	6 (16.2)	0.230
Cough > 3 months per year	4 (14.3)	5 (13.5)	1.000
Morning phlegm in winter	1 (3.6)	8 (21.6)	0.067
Coughing up phlegm throughout the day in winter	2 (7.1)	5 (13.5)	0.689
Phlegm > 3 months per year	0 (0)	6 (16.2)	0.033
Having a period of cough and phlegm lasting > 3 weeks in the past 3 years	7 (25.0)	8 (21.6)	0.749
Having > 1 periods of cough and phlegm lasting > 3 weeks in the past 3 years	1 (3.6)	7 (18.9)	0.124
Breathlessness	3 (10.7)	7 (18.9)	0.495
Wheezing in the past one year	0 (0)	2 (5.4)	0.502
Shortness of breath with wheezing	0 (0)	2 (5.4)	0.502
Spirometry testing			
FVC, mean ± SD, % of prediction	98.3 ± 10.8	88.9 ± 12.3	0.002
FEV1, mean ± SD, % of prediction	90.5 ± 8.6	79.4 ± 10.8	< 0.0001
FEV1/FVC, mean ± SD, %	84.3 ± 5.6	81.1 ± 7.1	0.053
Obstructive, n (%)	0 (0)	6 (16.2)	0.033
Restrictive, n (%)	1 (3.6)	6 (16.2)	0.130
Obstructive or restrictive, n (%)	1 (3.6)	12 (32.4)	0.004

**Table 4:** Logistic regression analysis for the association between cumulative respiratory silica exposure and ventilatory disorders

	Cumulative RCS exposure (mg/m^3^ x year)			Weighted tenure (year)		
	aOR	95% C.I.	*p* value	aOR	95% C.I.	*p* value
Using LLN as the cutoff values for FEV1/FVC						
Obstructive(LLN)	1.07	(0.98–1.18)	0.133	1.06	(0.99–1.12)	0.09
Restrictive(LLN)	1.21	(0.98–1.50)	0.081	1.05	(0.99–1.11)	0.124
Obstructive(LLN) or restrictive(LLN)	1.27	(1.03–1.56)	0.022	1.04	(1.01–1.09)	0.026
Using 0.75 as the cutoff value for FEV1/FVC						
Obstructive(0.75)	1.05	(0.96–1.16)	0.296	1.02	(0.97–1.07)	0.41
Restrictive(0.75)	1.21	(0.98–1.50)	0.081	1.04	(0.99–1.11)	0.124
Obstructive(0.75) or restrictive(0.75)	1.17	(1.01–1.35)	0.031	1.03	(0.99–1.07)	0.096
Using 0.70 as the cutoff value for FEV1/FVC						
Obstructive(0.70)	0.99	(0.77–1.28)	0.952	1.14	(0.94–1.37)	0.187
Restrictive(0.70)	1.21	(0.98–1.50)	0.081	1.05	(0.99–1.11)	0.124
Obstructive(0.70) or restrictive(0.70)	1.13	(0.98–1.32)	0.096	1.06	(1.00-1.12)	0.036

1. Obstructive: FEV1/FVC < cutoff values (LLN, 0.75, or 0.70)

2. Restrictive: FVC < LLN & FEV1/FVC > = cutoff values (LLN, 0.75, or 0.70)

3. Models were adjusted for age, sex, body mass index, education, current smoking, ex-smoking, and cumulative smoking amount

**Table 5:** Linear regression analysis for the association between cumulative respiratory silica exposure and lung function indices

	Cumulative RCS exposure (mg/m^3^ x year)			Weighted tenure (year)		
	Coefficient	95% C.I.	*P* value	Coefficient	95% C.I.	*P* value
FVC, % of prediction	-0.46	(-0.88–0.05)	0.03	-0.11	(-0.29-0.07)	0.238
FEV1, % of prediction	-0.45	(-0.81–0.08)	0.018	-0.12	(-0.28-0.05)	0.155
FEV1/FVC, %	-0.006	(-0.24-0.23)	0.957	-0.03	(-0.13-0.07)	0.592

Models were adjusted for age, sex, body mass index, education, current smoking, ex-smoking, and cumulative smoking amount

**Table 6:** Sensitivity analysis of the association between cumulative respiratory silica exposure and ventilatory disorders using logistic regression

	Cumulative RCS exposure (mg/m^3^ x year)			Weighted tenure (year)		
	aOR	95% C.I.	*p* value	aOR	95% C.I.	*p* value
Model 1						
Obstructive	1.08	(0.99–1.17)	0.086	1.03	(0.99–1.08)	0.122
Restrictive	1.09	(0.99–1.20)	0.065	1.03	(0.99–1.08)	0.105
Obstructive or restrictive	1.25	(1.05–1.49)	0.013	1.04	(1.01–1.08)	0.019
Model 2						
Obstructive	1.08	(0.99–1.18)	0.075	1.05	(1.00-1.11)	0.056
Restrictive	1.09	(0.99–1.20)	0.067	1.03	(0.99–1.08)	0.106
Obstructive or restrictive	1.27	(1.06–1.52)	0.011	1.05	(1.01–1.10)	0.01
Model 3						
Obstructive	1.08	(0.99–1.19)	0.072	1.06	(1.00-1.12)	0.049
Restrictive	1.09	(0.99–1.20)	0.089	1.03	(0.98–1.08)	0.182
Obstructive or restrictive	1.26	(1.06–1.51)	0.011	1.05	(1.01–1.10)	0.012
Model 4 (Never smokers)						
Obstructive	1.00	(0.82–1.22)	0.970	-	-	-
Restrictive	1.10	(0.90–1.35)	0.339	1.02	(0.96–1.08)	0.587
Obstructive or restrictive	1.13	(0.89–1.43)	0.320	1.07	(1.00-1.13)	0.041

1. Obstructive: FEV1/FVC < LLN

2. Restrictive: FVC < LLN & FEV1/FVC > = LLN

3. Analyses of Model1 were adjusted for age, sex, body mass index

4. Analyses of Model2 were adjusted for age, sex, body mass index, education

5. Analyses of Model3 were adjusted for age, sex, body mass index, education, current smoking

6. Analyses of Model4 only included never smokers and were adjusted for age, sex, body mass index, education

**Table 7:** Sensitivity analysis of the association between cumulative respiratory silica exposure and lung function indices using linear regression

	Cumulative RCS exposure (mg/m3 x year)			Weighted tenure (year)		
	Coefficient	95% C.I.	*p* value	Coefficient	95% C.I.	*p* value
Model1						
FVC, % of prediction	-0.43	(-0.83–0.03)	0.037	-0.11	(-0.26-0.05)	0.188
FEV1, % of prediction	-0.46	(-0.81–0.11)	0.01	-0.13	(-0.27-0.01)	0.059
FEV1/FVC, %	-0.04	(-0.26-0.19)	0.748	-0.04	(-0.12-0.05)	0.403
Model2						
FVC, % of prediction	-0.4	(-0.80-0.00)	0.051	-0.08	(-0.25-0.09)	0.352
FEV1, % of prediction	-0.43	(-0.77–0.08)	0.016	-0.11	(-0.25-0.04)	0.154
FEV1/FVC, %	-0.03	(-0.26-0.19)	0.788	-0.03	(-0.12-0.06)	0.469
Model3						
FVC, % of prediction	-0.39	(-0.80-0.02)	0.059	-0.07	(-0.25-0.11)	0.417
FEV1, % of prediction	-0.43	(-0.79–0.08)	0.016	-0.11	(-0.27-0.04)	0.146
FEV1/FVC, %	-0.04	(-0.27-0.19)	0.737	-0.04	(-0.14-0.05)	0.359
Model 4 (Never smokers)						
FVC, % of prediction	-0.60	(-1.21-0.01)	0.052	-0.05	(-0.31-0.21)	0.709
FEV1, % of prediction	-0.49	(-0.99-0.01)	0.054	-0.12	(-0.33-0.10)	0.272
FEV1/FVC, %	0.06	(-0.27-0.39)	0.722	-0.09	(-0.22-0.05)	0.198

1. Analyses of Model 1 were adjusted for age, sex, body mass index

2. Analyses of Model 2 were adjusted for age, sex, body mass index, education

3. Analyses of Model 3 were adjusted for age, sex, body mass index, education, current smoking

4. Analyses of Model 4 only included never smokers and were adjusted for age, sex, body mass index, education

A total of 20 participants, including one from the low-exposure group and 19 from the high-exposure group, underwent DLco and chest imaging at a hospital. Supplementary Table 2 S provides a comprehensive summary of the test data for these participants. Of these 20 participants, nine showed mild DLco reduction and nine had abnormal spirometry, with five of those with normal spirometry also showing DLco reduction. DLco negatively correlated with round and ground glass opacities (Spearman correlation − 0.529 and − 0.488, with *P*-values of 0.016 and 0.029, respectively). Linear regression indicated a -3.52% (95% CI, -6.73–0.30) reduction in DLco per 1 mg/m³ current RCS exposure increase (Table 8).

**Table 8:** Linear regression analysis for the association between current and cumulative respiratory silica exposure and diffusion capacity of lung

	Current RCS exposure (mg/m^3^)			Cumulative RCS exposure (mg/m^3^-year)		
	Coefficient	95% C.I.	*P* value	Coefficient	95% C.I.	*P* value
Dlco % of prediction	-3.52	(-6.73–0.30)	0.035	-0.44	(-0.91-0.04)	0.067
Dlco/VA % of prediction	-2.19	(-5.85-1.46)	0.214	-0.23	(-0.76-0.299)	0.359

Models were adjusted for age, sex, body mass index, education, current smoking, ex-smoking, and cumulative smoking amount

In the high-exposure group (S2 ∼ S20 in Supplementary Table 2 S), 63% (12 out of 19) showed CT abnormalities exceeding one grading score of ICOERD, while only 11% (2 of 19) had abnormalities on CXR, highlighting CT’s greater sensitivity. CT findings included rounded, irregular, and ground glass opacities, emphysema, large opacities, and subpleural lines. Among those with CT opacities, the lowest cumulative RCS exposure concentration was 0.67 mg/m³-year (median = 6.84, range = 0.67–44.4, interquartile range = 1.09–21.09). The shortest employment duration was 3 years (median = 5, range = 3–16, interquartile range = 4–6), and the shortest weighted tenure was 12 years (median = 36, range = 12–60, interquartile range = 21–48).

Table 9 shows the sensitivity and specificity of clinical methods for detecting CT opacities as defined by ICOERD. In predicting all CT opacities, symptom assessment alone had 58.3% sensitivity and 75% specificity, while spirometry showed higher sensitivity (66.7%) and specificity (87.5%). CXR had the lowest sensitivity (16.7%) but 100% specificity. DLco had 41.7% sensitivity and 50% specificity. Combining symptom assessment and spirometry reached the highest sensitivity (91.7%) with 75% specificity. Adding DLco maintained the same sensitivity but lowered specificity to 37.5%. Sensitivity for predicting round opacities was similar at 88.9%, with a slightly higher sensitivity for detecting ground-glass opacities at 100%.

**Table 9:** Sensitivity and specificity of various combinations of clinical methods as comparing to silicosis defined as chest CT detected opacities

	All CT opacities		Only RO		Only GGO	
	Sensitivity	Specificity	Sensitivity	Specificity	Sensitivity	Specificity
Any clinical abnormality						
Symptom	58.3	75.0	44.4	54.6	66.7	72.7
Spirometry	66.7	87.5	66.7	72.7	66.7	72.7
CXR	16.7	100	22.2	100	22.2	100
Dlco	41.7	50	55.6	63.6	55.6	63.6
Symptom + Spirometry	91.7	75.0	88.9	54.6	100	63.6
Spirometry + CXR	75.0	87.5	77.8	72.7	77.8	72.7
Spirometry + Dlco	75.0	37.5	77.8	36.4	77.8	36.4
Symptom + Spirometry + CXR	91.7	75.0	88.9	54.6	100	63.6
Symptom + Spirometry + Dlco	91.7	37.5	88.9	27.3	100	36.4
Symptom + Spirometry + CXR + Dlco	91.7	37.5	88.9	27.3	100	36.4

All CT opacities includes round, irregular, ground glass, and large opacities

RO: round opacities; GGO: ground glass opacities

## Discussion

This study establishes the association between cumulative exposure to respirable crystalline silica in artificial stone workers and lung function and imaging changes. Increased exposure leads to more ventilatory dysfunction and reduced lung function and DLco. Among 19 highly exposed workers, 63% showed CT opacities and 53% had employment duration less than 10 years (3 years in the shortest one), indicating possible accelerated silicosis. CT findings included nodules, ground glass opacities, and interstitial lines. Combining respiratory symptoms, lung function, and chest X-ray provided good sensitivity (91.7%) and specificity (75%) for the prediction of chest CT abnormalities. Weighting factors from questionnaires, used to estimate cumulative exposure, correlated well with exposure levels and lung function impacts.

Our research highlights considerable RCS exposure during raw material mixing, with a mean 8-hour TWA of 2.878 mg/m³, paralleling exposures seen in artificial stone fabrication using handheld power tools under dry conditions (approximately 1.3 to 3.9 mg/m³) [45]. In contrast, operations involving grinding and cutting machines in our study, in which wet methods were used, showed exposure levels (0.168 to 0.767 mg/m³) aligning with those in workshops using both dry and wet techniques (around 0.1 to 1.0 mg/m³) [45, 46]. A prior simulation of workplace conditions revealed that dry cutting artificial stone with a handheld circular saw could generate RCS concentrations up to 44.6 mg/m³. Introducing water to wet the saw blade brought this down tenfold to 4.934 mg/m³, and further reductions to 0.604 mg/m³ were achieved by combining the use of a wetted blade with local exhaust ventilation [47].

The mean RCS exposure level observed in this artificial stone manufacturing industry, at 0.647 mg/m³, surpasses those reported in other industries. For instance, in Iran, occupational RCS exposure levels have been documented to range from 0.12 mg/m³ in glass manufacturing to 0.24 mg/m³ in cement manufacturing, 0.25 mg/m³ in both asphalt manufacturing and brick production [48]. Meanwhile, in Denmark, average RCS exposures were recorded at 0.013 mg/m³ in fabricated metal products manufacturing, 0.069 mg/m³ in basic metals manufacturing, and 0.072 mg/m³ in the manufacturing of non-metallic mineral products [49]. Similarly, in Italy, mean RCS exposure levels were noted at 0.013 mg/m³ in basic metals manufacturing and 0.053 mg/m³ in the manufacturing of non-metallic mineral products [50].

In this study, silica exposure correlated with lung function decline, with a 0.45% (20 ml) FEV1 decrease per mg/m³-year increase, similar to Möhner et al.‘s uranium mining study [51]. While the mean RCS exposure (0.074 mg/m³) and cumulative RCS exposure (0.579 mg/m³-year) in Möhner et al.‘s research were lower than those in our study, the employment duration in their study (12.8 years) was twice as long as in ours (6 years). This discrepancy indicates a more rapid pulmonary response in situations of elevated exposure. The decline rate equalled the lung impact of smoking two pack-years [52]. Notably, after quitting smoking, the annual FEV1 decline decreased from 40 ml to 35 ml per year [53]. In contrast, even after exposure cessation, artificial stone silicosis led to a greater annual FEV1 decline (53–113 ml) [8] than the decline observed in non-smokers (30 ml) [53].

The logistic regression showed increasing risk trend for either obstructive or restrictive ventilatory defects with more silica exposure, affecting FVC and FEV1 but not the FEV1/FVC ratio. This suggests combined effects on both airways and lung parenchyma. Studies with lower exposure (0.57 mg/m^3^-year in average) [54] or without silicosis radiographic changes [55] linked silica exposure to FEV1/FVC ratio reduction. Our study’s higher exposure levels likely resulted in more interstitial lung changes, thereby highlighting the role of restrictive ventilatory defects. In the subgroup analysis, the association between obstructive ventilatory response and RCS exposure was not observed in workers who had never smoked. This suggests that the observed obstructive effect may not be directly attributable to RCS exposure but could be influenced by smoking, exposure to non-silica dust, or the synergistic effects of smoking and dust exposure.

DLco negatively correlated with the sum scores of round and ground glass opacities, significantly lower in workers exhibiting large opacities. Previous studies link severe silicosis-related CT abnormalities to lower DLco [56], especially with progressive massive fibrosis [10]. Recent research used work tenure to predict exposure to engineered stone dust, finding DLco abnormalities more likely with longer tenure [10, 19]. Our study shows a clear link between silica exposure and DLco, suggesting its potential as a biomarker for artificial stone dust exposure.

Chest CT, with its high sensitivity for detecting lung changes, is considered suitable for the early diagnosis of silicosis [31]. The importance of early detection of silicosis is highlighted by León-Jiménez et al.‘s study, which found that artificial stone workers, initially diagnosed with ILO category 0 silicosis, exhibited progression to categories 1 (42%) and 2 (21%), and some even to PMF (5%), within four years of ceasing exposure [8]. Patients with ILO category 0 silicosis present normal CXR but show relevant opacities on chest CT. The diagnosis of silicosis relies on documented exposure history and relevant imaging findings. Workers exposed to RCS who have normal CXR results during health screenings are not typically diagnosed with silicosis, despite possibly exhibiting mild respiratory symptoms or pulmonary function abnormalities. Consequently, this often results in unawareness of occupational health risks and a lack of practical industrial hygiene and preventive measures. However, the possibility of false positive chest CT opacities should be considered [57]. Non-occupational lung diseases can mimic the CT imaging characteristics of artificial stone silicosis; for instance, tuberculosis and sarcoidosis might present with small round opacities and lymph node enlargement, while hypersensitivity pneumonitis and infections could lead to ground-glass opacities, and autoimmune diseases might manifest as irregular opacities [29]. Therefore, an accurate diagnosis of silicosis relies on a comprehensive clinical assessment and careful differential diagnosis of CT imaging findings.

In this study, 63% of highly exposed workers had abnormal CT opacities, similar to a Spanish family-owned factory survey [7]. In contrast, a U.S. study using chest X-rays reported a 12% prevalence [24]. The lowest observed adverse effect level for CT abnormalities in this study was 0.168 mg/m^3^ and 0.672 mg/m^3^-year, suggesting the utility of CT above this exposure level. However, an Israeli study highlighted silicosis cases at even lower exposures (0.02 mg/m³ and 0.34 mg/m³-year) [58], indicating potential risks at lower levels. Consequently, the implementation of CT screening necessitates a careful evaluation of its costs, associated risks of radiation and unnecessary medical intervention, and the added diagnostic value. This underscores the need for further research to assess the cost-effectiveness of employing chest CT screening among workers exposed to high RCS levels.

The utility of spirometry and chest X ray in the monitoring workers with lower levels of AS exposure remains to be demonstrated [27]. Our analysis of sensitivity and specificity suggests that when aiming to detect chest CT abnormalities in workers with high exposure, priority could be given to those with abnormalities identified through respiratory questionnaires, spirometry, or CXR. A recent study categorized simple silicosis based on the presence of round opacities on chest CT in artificial stone workers [27]. In our research, 9 out of 19 (47%) workers with high exposure exhibited round opacities on chest CT, which could be considered as more definitive cases of simple silicosis. The accuracy of combined results from other clinical methods showed similar precision in identifying all types of opacities and specifically round opacities. The addition of DLco measurement did not enhance predictive accuracy and even reduced specificity. This reduction in specificity may be related to the inherently higher test variability associated with diffusion capacity assessments. Small inaccuracies in measuring inspiratory flows or exhaled gas concentrations can lead to significant errors in DLco [36]. Normal variations in DLco can reach approximately 7% within a day and up to 19% from week to week [36].

Glass et al. created a questionnaire-based method to assign weighting factors for estimating cumulative silica exposure [19]. They linked increased weighted tenure to higher risks for dyspnea and abnormalities in chest X-ray, spirometry, and DLco. Our study confirms a significant correlation between weighted tenure and both silica exposure and lung function abnormalities. This supports using weighting factors in large-scale epidemiological studies or respiratory health monitoring.

Following surveillance, industrial hygiene control measures were implemented, including training on proper respiratory PPE use, fit testing, enhancing ventilation for quartz storage and mixers, isolating mixing areas, and relocating control panels outside these areas. The participants received personal health reports and guidance concerning health findings, including referral to nearby medical facilities if desired. Workers’ rights were also communicated. In Taiwan, the workers with occupational diseases are protected under the Labor Occupational Accident Insurance and Protection Act, which allows them to seek compensation for medical expenses, sick leave, disability, and mortality. This regulation also prohibits employers from dismissing workers who are diagnosed with occupational diseases.

Our study’s strengths lie in combining silica exposure assessment and health surveillance, and recruiting workers with varying exposure levels, enhancing the exposure-response relationship analysis. Conducting chest CT scans on highly exposed workers helped identify early lung changes due to silica and evaluate the efficacy of different clinical methods for diagnosing silica-related respiratory effects.

Our study’s small sample size limits its representativeness to all artificial stone workers. Unlike prior studies on slab fabricators and countertop installers, we focused on artificial stone manufacturing, examining high exposure in dry stone dust and resin mixing, and moderate exposure in wet slab cutting and grinding. This unique focus highlights the unexplored health impacts in this sector. Additionally, our cross-sectional design limits causal conclusions. Despite controlling for factors like smoking habits and cumulative exposure, the healthy worker effect may have underestimated the exposure-response relationship.

In conclusion, our study links silica exposure in artificial stone manufacturing to decreased lung function and more ventilatory abnormalities, with abnormal chest CT opacities frequent in highly exposed workers. Individuals with positive respiratory questionnaires, spirometry, or chest X-rays could be considered a priority for chest CT. The short exposure time and low lowest observable adverse effect level (LOAEL) for CT abnormalities highlights the need for early CT scanning and ongoing health monitoring. Where workplace silica data is lacking, questionnaire-based weighting factors may serve as a surrogate for exposure assessment.

### Electronic supplementary material

Below is the link to the electronic supplementary material.

Supplementary Material 1

## Data Availability

No datasets were generated or analysed during the current study.

## Abbreviations

aOR: Adjusted odds ratios
AS: Artificial stone
CT: Computed tomography
CXR: Chest X-ray
DLco: Diffusion capacity for carbon monoxide
EM: Emphysematous changes
FEV1: Forced expiratory volume in one second
FVC: Forced vital capacity
GGO: Ground-glass opacities
ICOERD: The International Classification of High-Resolution Computed Tomography for Occupational and Environmental Respiratory Diseases
IR: Linear and/or irregular opacities,
LLN: Lower limit of normal
RCS: Respirable crystalline silica
RO: Well-defined rounded opacities,
RPE: Respiratory protective equipment
TWA: Time-weighted average
WF: Weighting factor
