# Supplementary material for: Dose-response relationship between lung function and chest imaging response to silica exposures in artificial stone manufacturing workers
Source: Environ Health. 2024 Mar 2;23:25. doi: 10.1186/s12940-024-01067-1 (PMC10908069; doi:10.1186/s12940-024-01067-1)
Supplement: Supplementary file 1 — Supplementary Material 1 [file 12940_2024_1067_MOESM1_ESM.docx]

Supplementary table 1S. Comparison of basic characteristics, job exposure, and health outcomes among workers across two manufacturing plants

|  |  | Plant A | Plant B |  |
| --- | --- | --- | --- | --- |
|  | N | n=57 | n=8 | p value |
| Age, mean ± SD, yr | 65 | 41.50 ± 11.98 | 42.76 ± 8.52 | 0.776 |
| Male, n (%) | 65 | 45 (78.9) | 6 (75.0) | 1.000 |
| Body mass index, mean ± SD | 65 | 25.35 ± 4.65 | 28.48 ± 3.86 | 0.075 |
| Education attainment>=13 yrs, n (%) | 65 | 39 (68.4) | 2 (25.0) | 0.044 |
| Tobacco smoking, n (%) | 65 |  |  | 0.956 |
| Never | 39 | 34 (59.7) | 5 (62.5) |  |
| Ex-smoker | 7 | 6 (10.5) | 1 (12.5) |  |
| Current smoker | 19 | 17 (29.8) | 2 (25.0) |  |
| Cumulative smoking amount, mean ± SD, pack*yr | 65 | 7.42 ± 8.64 | 11.24 ± 11.54 | 0.442 |
| Tenure, year, mean ± SD | 65 | 5.88 ± 5.58 | 6.57 ± 4.73 | 0.739 |
| Weighting factor, mean ± SD | 65 | 3.56 ± 3.22 | 2.81 ± 2.15 | 0.524 |
| Weighted tenure, mean ± SD, year | 65 | 18.05 ± 19.89 | 18.61 ± 18.71 | 0.939 |
| Cumulative RCS exposure, mean ± SD, mg/m^3^*year | 65 | 3.70 ± 7.87 | 2.96 ± 3.26 | 0.795 |
| Best respiratory PPE used at work | 65 |  |  | 0.628 |
| No or regular flat mask, n (%) | 4 | 4 (7.0) | 0 (0) |  |
| N95, n (%) | 23 | 19 (33.3) | 4 (50.0) |  |
| Half-face mask, n (%) | 12 | 10 (17.5) | 2 (25.0) |  |
| Full-face mask, n (%) | 17 | 15 (26.3) | 2 (25.0) |  |
| PAPR, n (%) | 9 | 9 (15.8) | 0 (0) |  |
| Fit test experience, n (%) | 65 | 7 (12.3) | 1 (12.5) | 0.986 |
| Job title | 65 |  |  | 0.018 |
| Administration | 15 | 15 (26.3) | 0 (0) |  |
| Research and Development | 4 | 4 (7.0) | 0 (0) |  |
| Quality Control | 2 | 2 (3.5) | 0 (0) |  |
| Vacuum Press Machine Operator | 4 | 4 (7.0) | 0 (0) |  |
| Facility Management | 15 | 13 (22.8) | 2 (25.0) |  |
| Grinding Machine Operator | 8 | 5 (8.8) | 3 (37.5) |  |
| Operations Supervisor | 4 | 3 (5.3) | 1 (12.5) |  |
| Cutting Machine Operator | 3 | 1 (1.8) | 2 (25.0) |  |
| Raw Material Operator | 10 | 10 (17.5) | 0 (0) |  |
| Respiratory symptoms, n (%) |  |  |  |  |
| Morning cough in winter | 65 | 9 (15.8) | 1 (12.5) | 1.000 |
| Coughing throughout the day in winter | 65 | 12 (21.1) | 2 (25.0) | 1.000 |
| Cough > 3 months per year | 65 | 8 (14.0) | 1 (12.5) | 1.000 |
| Morning phlegm in winter | 65 | 8 (14.0) | 1 (12.5) | 1.000 |
| Coughing up phlegm throughout the day in winter | 65 | 6 (10.5) | 1 (12.5) | 1.000 |
| Phlegm > 3 months per year | 65 | 5 (8.8) | 1 (12.5) | 0.561 |
| Having a period of cough and phlegm lasting > 3 weeks in the past 3 years | 65 | 14 (24.6) | 1 (12.5) | 0.669 |
| Having > 1 periods of cough and phlegm lasting > 3 weeks in the past 3 years | 65 | 7 (12.3) | 1 (12.5) | 1.000 |
| Breathlessness | 65 | 9 (15.8) | 1 (12.5) | 1.000 |
| Wheezing in the past one year | 65 | 1 (1.8) | 1 (12.5) | 0.233 |
| Shortness of breath with wheezing | 65 | 2 (3.5) | 0 (0) | 1.000 |
| Spirometry testing |  |  |  |  |
| FVC, mean ± SD, % of prediction | 65 | 93.0 (12.4) | 92.5 (14.4) | 0.922 |
| FEV1, mean ± SD, % of prediction | 65 | 84.0 (10.9) | 85.6 (14.5) | 0.715 |
| FEV1/FVC, mean ± SD, % | 65 | 82.4 (6.9) | 83.0 (5.0) | 0.819 |
| Obstructive, n (%) | 65 | 6 (10.5) | 0 (0) | 1.000 |
| Restrictive, n (%) | 65 | 6 (10.5) | 1 (12.5) | 1.000 |
| Obstructive or restrictive, n (%) | 65 | 12 (21.1) | 1 (12.5) | 1.000 |
| Chest CT abnormality | 20 | 10 (62.5) | 2 (50.0) | 1.000 |
| Dlco, % of prediction | 20 | 72.3 (11.1) | 81.4 (3.7) | 0.129 |
| Dlco/VA, % of prediction | 20 | 80.9 (9.5) | 89.3 (9.9) | 0.139 |
| Diffusion capacity impairment | 20 | 9 (56.3) | 0 (0) | 0.094 |

P values were determined using Fisher's exact test and Student's t-test.

Supplementary table 2S. Summary of chest CT and lung function results in 20 workers

| Subject | RCS exposure (mg/m3) | WF | Tenure | Chest CT description | RO sum grades | IR sum grades | GGO sum grades | EM sum grades | LO type | CXR abnormality | FEV1% | FVC% | FEV1/FVC | Dlco% | Spirometry disorder | Dlco disorder |
| --- | --- | --- | --- | --- | --- | --- | --- | --- | --- | --- | --- | --- | --- | --- | --- | --- |
| S1 | 0.00133 | 1 | 20 | A tiny nodule at LML | 0 | 0 | 0 | 0 | 0 | No | 87.5 | 85 | 0.95 | 63.2 | No | Mild |
| S2 | 0.587 | 3 | 5 | Negative | 0 | 0 | 0 | 0 | 0 | No | 83 | 95.9 | 0.8 | 83.7 | No | No |
| S3 | 0.2 | 6 | 4 | Mild interseptal thickening at bilateral lower lung | 0 | 3 | 0 | 0 | 0 | No | 91.8 | 123.3 | 0.68 | 85.6 | Obstructive | No |
| S4 | 0.622 | 3 | 16 | Scattered ground glass nodules at upper lung; upper lobe bullae | 1 | 0 | 1 | 1 | 0 | No | 55.2 | 64.9 | 0.77 | 76 | Restrictive | No |
| S5 | 0.587 | 0.8 | 9 | Solitary lung nodule | 0 | 0 | 0 | 0 | 0 | No | 88 | 87.2 | 0.84 | 82.1 | No | No |
| S6 | 0.2 | 7.5 | 5 | Negative | 0 | 0 | 0 | 0 | 0 | No | 70.6 | 82.6 | 0.76 | 81.8 | No | No |
| S7 | 0.168 | 7.5 | 6 | Scattered nodules at upper lung | 2 | 0 | 0 | 0 | 0 | No | 95.3 | 99.7 | 0.87 | 83.9 | No | No |
| S8 | 4.44 | 6 | 10 | Mild ground glass opacities and interseptal thickening at bilateral lower lung | 0 | 2 | 2 | 0 | 0 | No | 73 | 78.5 | 0.85 | 83.2 | Restrictive | No |
| S9 | 4.44 | 8 | 6 | Bilateral upper lung fibronodular scaring and ground glass opacities; large opacity at RUL; subpleural curvilinear lines at LUL; focal tree-in-bud | 2 | 0 | 3 | 0 | A | Yes | 62.7 | 67.1 | 0.87 | 52.3 | Restrictive | Mild |
| S10 | 4.44 | 8 | 4 | Scattered nodules at bilateral upper lung; focal air trapping | 3 | 0 | 0 | 0 | 0 | No | 76.3 | 93 | 0.75 | 75.1 | Obstructive | No |
| S11 | 4.44 | 8 | 3 | Focal ground glass opacities at RUL and LLL; focal air trapping at bilateral lung; scattered nodules; subpleural curvilinear lines at RUL and RML | 4 | 0 | 2 | 0 | 0 | No | 65.9 | 85.9 | 0.7 | 60.1 | Obstructive | Mild |
| S12 | 0.622 | 8 | 6 | Scattered nodules at bilateral upper lung; upper lobe predominant ground glass opacities | 2 | 2 | 3 | 0 | 0 | No | 85.5 | 87.2 | 0.89 | 76.1 | No | No |
| S13 | 4.44 | 4 | 5 | Scattered nodules and ground glass opacities at bilateral upper lung; focal air trapping | 2 | 0 | 2 | 0 | 0 | No | 71.8 | 91.3 | 0.73 | 59.2 | Obstructive | Mild |
| S14 | 0.27 | 8 | 5 | Bilateral upper lung fibronodular scaring; bilateral ground glass opacities; large opacity with cavitation at RUL; subpleural curvilinear lines at bilateral upper lung | 4 | 2 | 8 | 5 | A | Yes | 76.3 | 83 | 0.83 | 65.9 | No | Mild |
| S15 | 0.587 | 8 | 7 | Solitary lung nodule at RUL | 0 | 0 | 0 | 0 | 0 | No | 78.6 | 89.2 | 0.74 | 91.5 | Obstructive | No |
| S16 | 0.168 | 7.5 | 9 | Parenchymal band at RML | 0 | 0 | 0 | 0 | 0 | No | 70.5 | 79.9 | 0.82 | 72.4 | No | Mild |
| S17 | 0.168 | 3 | 4 | Mild interseptal thickening at bilateral lower lung; mild ground glass opacity at LLL; parenchymal band at RLL | 0 | 4 | 1 | 0 | 0 | No | 99.8 | 104 | 0.83 | 83.6 | No | No |
| S18 | 0.767 | 6 | 3 | Scattered nodules at upper lung; focal air trapping; subpleural curvilinear lines at bilateral upper lung; ground glass opacities | 4 | 0 | 2 | 0 | 0 | No | 69.5 | 76 | 0.82 | 61.9 | Restrictive | Mild |
| S19 | 0.168 | 6 | 6 | Parenchymal band at RML | 0 | 0 | 0 | 0 | 0 | No | 88.5 | 105.6 | 0.76 | 73.3 | No | Mild |
| S20 | 0.767 | 3 | 8 | Negative | 0 | 0 | 0 | 0 | 0 | No | 82 | 86.2 | 0.87 | 71.2 | No | Mild |

Abbreviations: RCS, respirable crystalline silica; CXR, chest X-ray; FEV1%, percent of predicted forced expiratory volume at one second; FVC%, percent of predicted forced vital capacity; DLco%, percent of predicted diffusion capacity for carbon monoxide; RO, round opacity; IR, irregular opacity; GGO, ground glass opacity; EM, emphysema; LO, large opacity; RUL, right upper lung; RML, right middle lung; RLL, right lower lung; LUL, left upper lung; LML, left middle lung; LLL, left lower lung
